# A filled chocolates technique to seal negative-pressure wound therapy around external fixation devices: a randomized controlled trial

**DOI:** 10.1186/s13018-021-02747-1

**Published:** 2021-10-12

**Authors:** Wei Fan, Fushan Hou, Kun Xi, Chen Hao, Xiangdong Lu, Bin Zhao

**Affiliations:** 1 Department of Orthopedics, The Second Hospital of Shanxi Medical University, No. 382 Wuyi Road, Taiyuan, 030001 Shanxi People’s Republic of China; 2 Department of Orthopedics, The First Affiliated Hospital of Soochow University, 188 Shizi Road, Suzhou, 215006 Jiangsu People’s Republic of China

## Abstract

**Background:**

In complex injuries, external fixation device represents a challenge to maintain negative-pressure wound therapy (NPWT). In this trial, we compared a combination of bone wax and colostomy paste versus bone wax alone to seal NPWT around external fixation devices.

**Methods:**

Debridement surgeries of limbs with open fracture and large soft tissue defect need NPWT to be applied around the external fixation devices were randomized into two groups. The seal between external fixation devices and the drape was established using either bone wax first and then reinforced with colostomy paste or bone wax alone. The primary outcome was seal failure within 3 days of debridement. Secondary outcomes included the number of seal failure per debridement surgery and the time spent in repairing the seal within 3 days.

**Results:**

A total of 56 debridement surgeries were enrolled: 28 to the bone wax/colostomy paste group versus 28 to the bone wax control group. One patient in the control group died 1 day after the first debridement surgery. One patient in the bone wax/colostomy paste group was transferred to other hospitals within 3 days. The final analysis included 27 debridement surgeries in the bone wax/colostomy paste group and 27 debridement surgeries in the control group. The rate of seal failure (defined by loss of negative pressure at anytime within 3 days) was 81.5% (22/27) in the control group versus 11.1% (3/27) in the bone wax/colostomy paste group (*p* < 0.001; *χ*^2^ test). The bone wax/colostomy paste group also had significantly lower number of seal failures per debridement (median of 0 vs. 2; *p* = 0.004), and shorter time spent in repairing the seal (median of 0 vs. 18 min; *p* < 0.001).

**Conclusions:**

Using bone wax followed by colostomy paste to seal NPWT around external fixation devices reduces seal failure.

## Background

Negative-pressure wound therapy (NPWT), also known as vacuum-assisted closure (VAC) or vacuum sealing drainage (VSD), is commonly used to manage large soft tissue defect. NPWT promotes drainage, thus minimizes bacterial colonization [1]. It also reduces tissue edema, increases local perfusion, and stimulates granulation tissue formation [1]. The negative pressure environment alters the cytoskeleton of cells in the wound bed, and by doing so, increases fibroblast proliferation [2]. In patients with open fracture and large soft tissue defect, however, external fixation device may represent a challenge in maintaining a proper seal. A variety of methods, including the use of bone wax [3], are helpful but seal failure remains frequent. Colostomy paste has also been reported to seal the junction between the pin and plastic drape [4]. Colostomy paste has properties (high viscosity and plasticity) to maintain the seal, but is non-sterile, and thus could be a source of contamination upon entry into the wound due to negative pressure. We combined the 2 methods to maintain NPWT in patients with open fracture (Gustilo II, Gustilo III [5]) and large soft tissue defect. Briefly, seal was created using sterile bone wax between external fixation pins and the drape and then reinforced with colostomy paste.

## Methods

### Study design and patients

This trial was approved by the hospital Ethics Committee and is reported in line with the Consolidated Standards of Reporting Trials (CONSORT) Guidelines. Two attending physicians and a single radiologist reviewed all images and agreed on the classification of the fracture. All patients gave written informed consent prior to enrollment. Inclusion criteria included: (1) debridement surgeries of limbs with open fracture and large soft tissue defect need NPWT to be applied around the external fixation devices; (2) only two external fixation pins were present in the area of planned NPWT. The surgery and the sealing were conducted by a single orthopedic surgeon. The primary outcome was seal failure within 3 days of debridement. Secondary outcomes included the number of seal failure per debridement surgery and the time spent in repairing the seal within 3 days. This trial is registered at the official China Clinical Trial Registration site (CHiCTR1900028080).

### Vacuum seal

**Fig. 1:**
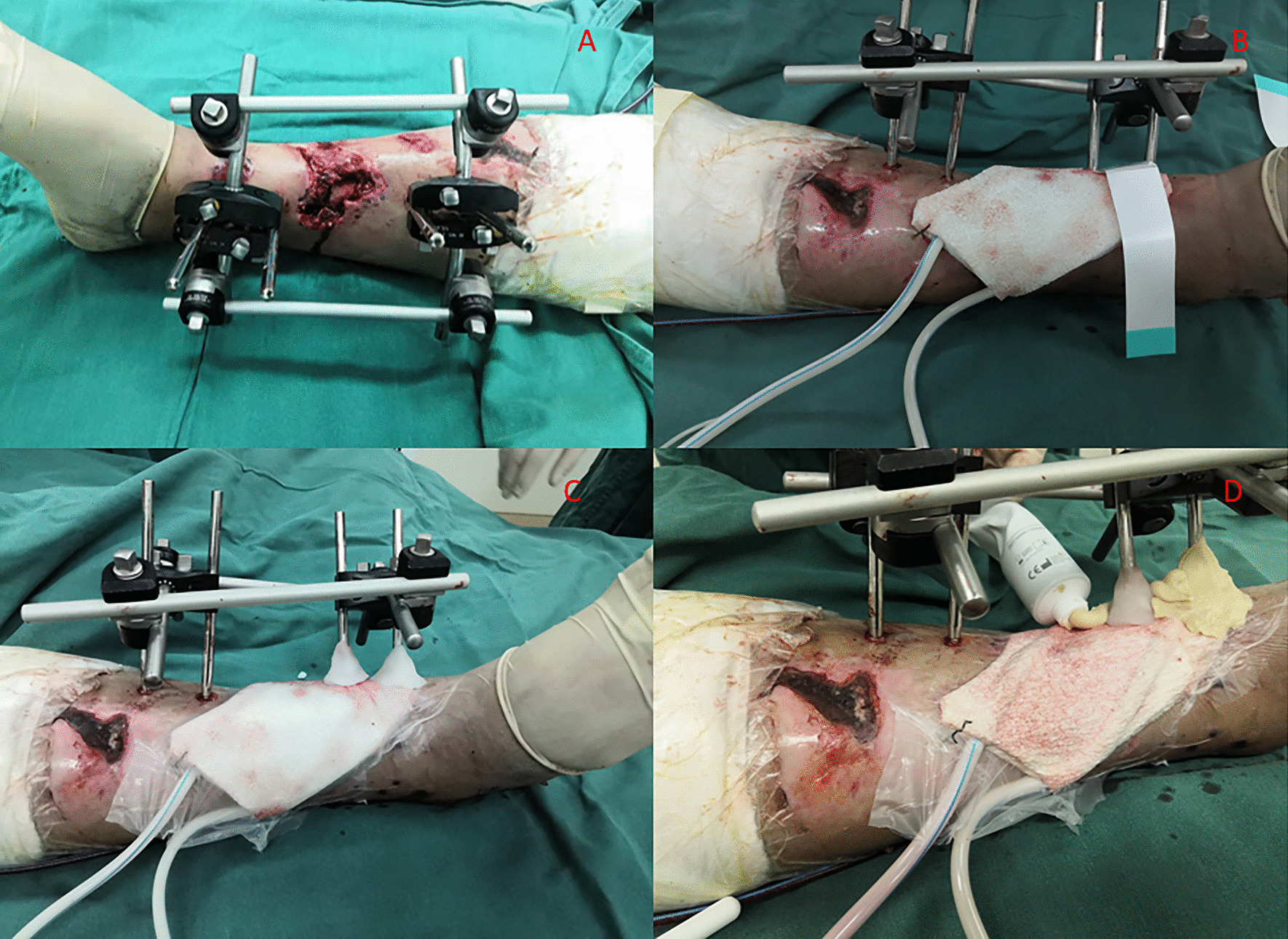
An operation process using the filled chocolate sealing technology: **A** External fixation bracket installation after wound debridement. **B** Sealing of the VSD semi-permeable membrane after suture placement. **C** Sterile bone wax creating a tight seal around the needle track and semi-permeable membrane. **D** Colostomy paste spread evenly around the bone wax structure

**Fig. 2:**
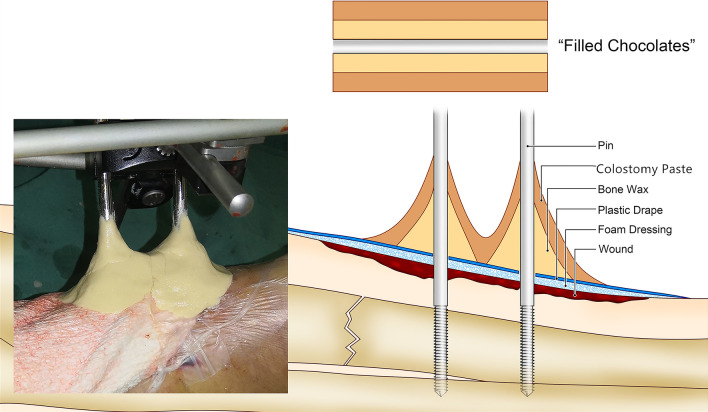
Schematic diagram of the composition of the "Filled Chocolates"

NPWT was conducted using a routine protocol. Briefly, sterile dressings were cut to fit the area of soft tissue defect and fixed to surrounding healthy skin using either staples or sutures. The surrounding oily skin and external fixation pins(Trauson Medical Instruments, China) were cleaned thoroughly before adhesive plastic drape was used to seal the dressings (and not onto the pins). The vacuum pump (Wuhan VSD Medical Science & Technology, China) was activated, and then, small amount of sterile bone wax was applied onto the plastic drape around the pins to obtain the vacuum. External fixation pins with different diameters (2.5 mm,3.0 mm,4.0 mm,5.0 mm) were used according to different fracture sites. In the bone wax/colostomy paste group, colostomy paste (Coloplast, Medical Products Sales, LTD, China) was applied to wrap the bone wax to reinforce the seal. Efforts were made so that colostomy paste was in close contact with both the plastic drape and external fixation screws (Fig. 1). This method is called “Filled Chocolates Technique” by our team (Fig. 2). The pressure was maintained at -125 mm Hg throughout the study period.

### Statistical analysis

Continuous variables were tested for normality using the Shapiro–Wilk test. Normally distributed variables are expressed as mean ± standard deviation, and group difference was examined using Student's *t* test. Variables not following normal distribution are reported as median and interquartile range (IQR), and analyzed using a rank-based nonparametric test. Categorical variables were analyzed using Pearson's *χ*^2^ test. *p* < 0.05 (2-sided) was considered statistically significant. All statistical analyses were conducted using the SPSS 22.0.

## Results

A total of 56 debridement surgeries of limbs were enrolled during a period from June 2018 to March 2020: 28 to the bone wax/colostomy paste group and remaining 28 to the bone wax control group. One patient in the control group died one day after the first debridement surgery due to severe head trauma. One patient in the bone wax/colostomy paste group was transferred to other hospitals two days after the first debridement surgery and lost to follow-up. The final analysis included 27 debridement surgeries in the bone wax/colostomy paste group and 27 debridement surgeries in the control group. The rate of seal failure (defined by loss of negative pressure at anytime within 3 days) was 81.5% (22/27) in the control group versus 11.1% (3/27) in the bone wax/colostomy paste group (*p* < 0.001; *χ*^2^ test) (Table 1). The number of seal failure was also lower in the bone wax/colostomy paste group (median of 0 vs. 2; *p* = 0.004) (Table 1). The time spent in repairing the seal was significantly shorter in the bone wax/colostomy paste group (median of 0 vs. 18 min; *p* < 0.001) (Table 1).

**Table 1:** Descriptive statistics of patients

	Bone wax/colostomy paste	Bone wax alone	*p*
	(*n* = 27)	(*n* = 27)	
Seal failure, y(%)	3 (11.1)	22 (81.5)	< 0.001^a^
Number of seal failures per debridement #, median(IQR)	0 (0–0)	2.0 (1.5–2.5)	= 0.004^b^
Time spent in repairing the seal minutes, median (IQR)	0 (0–0)	18 (9.5–24.5)	< 0.001^b^

^a^Pearson's *χ*^2^ test

^b^Rank-based nonparametric test

## Discussion

Patients who require external fixation represent a major challenge for NPWT, particularly when external fixation pins and screws are inside the area of intended NPWT. A variety of methods have been developed to create a reliable seal [3–12]. Incorporate the bulk of the external fixation device within the large ioband drapes or a separate piece of foam could secure NPWT [6, 7] but sometimes interferes with the assessment of skin condition and blood supply of the tissue. This method also interferes with the movement and functional exercise of the affected limb. There is also a risk that the pins and wires puncture the drape after the application of the suction [4]. Skin adhesives improve the adhesiveness of the plastic drape to the skin in maintaining the seal in difficult areas [8–10], but have not proven useful in sealing the gap between plastic drape and external fixation pins. Rubber strips have been reported to be tightly tied around the pins outside the adhesive plastic drape to obtain the vacuum [11], but the adhesive plastic drape remnants on the pins and screws are difficult to remove in subsequent debridement. Placing sterile bone wax at the junction between the screws and plastic drape is another method to create vacuum [3], but is prone to seal failure due to low viscosity and adhesiveness. Colostomy paste has also been reported to seal the junction between the screw and plastic drape [4]. Colostomy paste has desirable properties, including high viscosity and plasticity, but is non-sterile, and thus could contaminate the wound if entering the wound along pressure gradient. In the “Filled Chocolates Technique”, sterile bone wax was applied to seal the gap between external fixation pins and drape to obtain the vacuum; the seal was then reinforced by applying colostomy paste over the bone wax. This method takes advantage of the desirable property of colostomy paste while avoiding direct contact between the colostomy paste and the dressing, or even the wound. Furthermore, bone wax and colostomy paste can be easily removed from the pins and screws with an iodopovidone-soaked gauze for subsequent debridements. The results of this trial demonstrated a dramatic reduction in seal failure.

A major limitation in the current study was the relatively short observation period. Second, we did not examine whether the decrease in seal failure improves the treatment outcomes.

Using bone wax followed by colostomy paste to seal NPWT around external fixation devices could reduce seal failure. Whether this method could decrease the rate of infection and produce clinically meaningful results requires further studies.

## Data Availability

The datasets used and/or analysed during the current study are available from the corresponding author on reasonable request.

## Abbreviations

NPWT: Negative-pressure wound therapy
VAC: Vacuum-assisted closure
VSD: Vacuum sealing drainage
